# Nonspecific effects of oral vaccination with live-attenuated *Salmonella* Typhi strain Ty21a

**DOI:** 10.1126/sciadv.aau6849

**Published:** 2019-02-27

**Authors:** S. H. Pennington, D. M. Ferreira, E. Caamaño-Gutiérrez, J. Reiné, C. Hewitt, A. D. Hyder-Wright, S. B. Gordon, M. A. Gordon

**Affiliations:** 1 Department of Clinical Infection, Microbiology and Immunology, Institute of Infection and Global Health, University of Liverpool, Liverpool, UK.; 2 Department of Clinical Sciences, Liverpool School of Tropical Medicine, Liverpool, UK.; 3 Department of Parasitology, Liverpool School of Tropical Medicine, Liverpool, UK.; 4 Computational Biology Facility, Institute of Integrative Biology, University of Liverpool, Liverpool, UK.; 5 Malawi-Liverpool-Wellcome Trust Clinical Research Programme, P.O. Box 30096, Blantyre 3, Malawi.

## Abstract

Epidemiological and immunological evidence suggests that some vaccines can reduce all-cause mortality through nonspecific changes made to innate immune cells. Here, we present the first data to describe the nonspecific immunological impact of oral vaccination with live-attenuated *Salmonella* Typhi strain Ty21a. We vaccinated healthy adults with Ty21a and assessed aspects of innate and adaptive immunity over the course of 6 months. Changes to monocyte phenotype/function were observed for at least 3 months. Changes to innate and adaptive immune cell cytokine production in response to stimulation with vaccine and unrelated nonvaccine antigens were observed over the 6-month study period. The changes that we have observed could influence susceptibility to infection through altered immune responses mounted to subsequently encountered pathogens. These changes could influence all-cause mortality.

## Teaser

Vaccination with weakened strains of *Salmonella* may protect against other infections.

## INTRODUCTION

Epidemiological evidence has demonstrated that live-attenuated vaccines can reduce all-cause mortality (*1*, *2*). The strongest evidence has been collected in resource-poor settings after vaccination with oral polio vaccine (OPV), Bacille Calmette-Guérin (BCG), and measles-containing vaccines (*3*–*7*). Recently, the World Health Organization Special Advisory Group of Experts has recommended that further research should be undertaken to better understand the nonspecific impact of vaccination on all-cause mortality (*8*, *9*).

Nonspecific beneficial effects of vaccination are believed to be the result of the generation of innate immune memory through epigenetic modification (*10*–*12*). These changes may manifest themselves in the form of phenotypic variation among circulating innate cell populations, as well as altered cytokine production in response to in vitro cell stimulation (*13*).

Toll-like receptor 5 (TLR-5) engagement at the mucosal surface of the gastrointestinal tract is known to enhance immune responses to influenza vaccination (*14*). It has been demonstrated that the live-attenuated oral *Salmonella* vaccine, Ty21a, has the capacity to aid in the regression of bladder cancer, which is believed to be the result of TLR engagement (*15*). We have also observed enhanced cellular responses to influenza virus at the duodenal mucosa 18 days after vaccination with Ty21a (*16*). We therefore hypothesized that oral vaccination with Ty21a may have the capacity to generate innate immune memory and alter immune responses to unrelated pathogens.

A number of *Salmonella*-based vectors are in development (*17*, *18*) and an increased understanding of the off-target impacts of live-attenuated strains of *Salmonella* could further inform their development. The identification of bacteria that generate innate immune memory may lead to the development of therapeutics, vectors, and adjuvants capable of reducing all-cause mortality and/or enhancing the efficacy of vaccines targeting a wide array of unrelated pathogens.

We have previously shown that vaccination with Ty21a generates long-lived vaccine-specific peripheral immune responses (*19*). Here, we have assessed the wider, off-target impact of vaccination with Ty21a on human immunity at 14 days, 3 months, and 6 months after vaccination. We assessed innate immune cell surface marker expression as well as interferon-γ (IFN-γ) [T helper 1 (T_H_1)], interleukin-4 (IL-4) (T_H_2), IL-17A (T_H_17), transforming growth factor–β (TGF-β) (regulatory), and tumor necrosis factor–α (TNF-α) (T_H_1) production among B cells, CD4^+^ T cells, CD8^+^ T cells, monocytes, mucosal-associated invariant T (MAIT) cells, and γδ T cells after in vitro stimulation with a range of antigens.

## RESULTS

### Volunteer recruitment

Volunteers aged between 18 and 60 years were invited to participate in this study. Volunteers were excluded from participation if they had previously been immunized with Ty21a or if they had previously visited a typhoid-endemic region (Asia or sub-Saharan Africa). Volunteers were also excluded if they were pregnant, if they were taking any immunosuppressive medications, or if they had a chronic illness. There were no substantial differences between the groups with regard to age or gender; however, it should be noted that considerably more females than males were recruited to both the vaccinated group and the control group (Table 1).

**Table 1:** Volunteer information.

**Study group**	**Number of volunteers**	**Age (years)**	**Sex (M:F)**	**BCG (Y:N)**	**Study period**
Control	14	36 ± 13.3	2:12	9:5	May 2015–December 2015
Vaccination	16	32 ± 10.1	4:12	13:3	June 2015–December 2015

### Monocyte phenotype

**Fig. 1:**
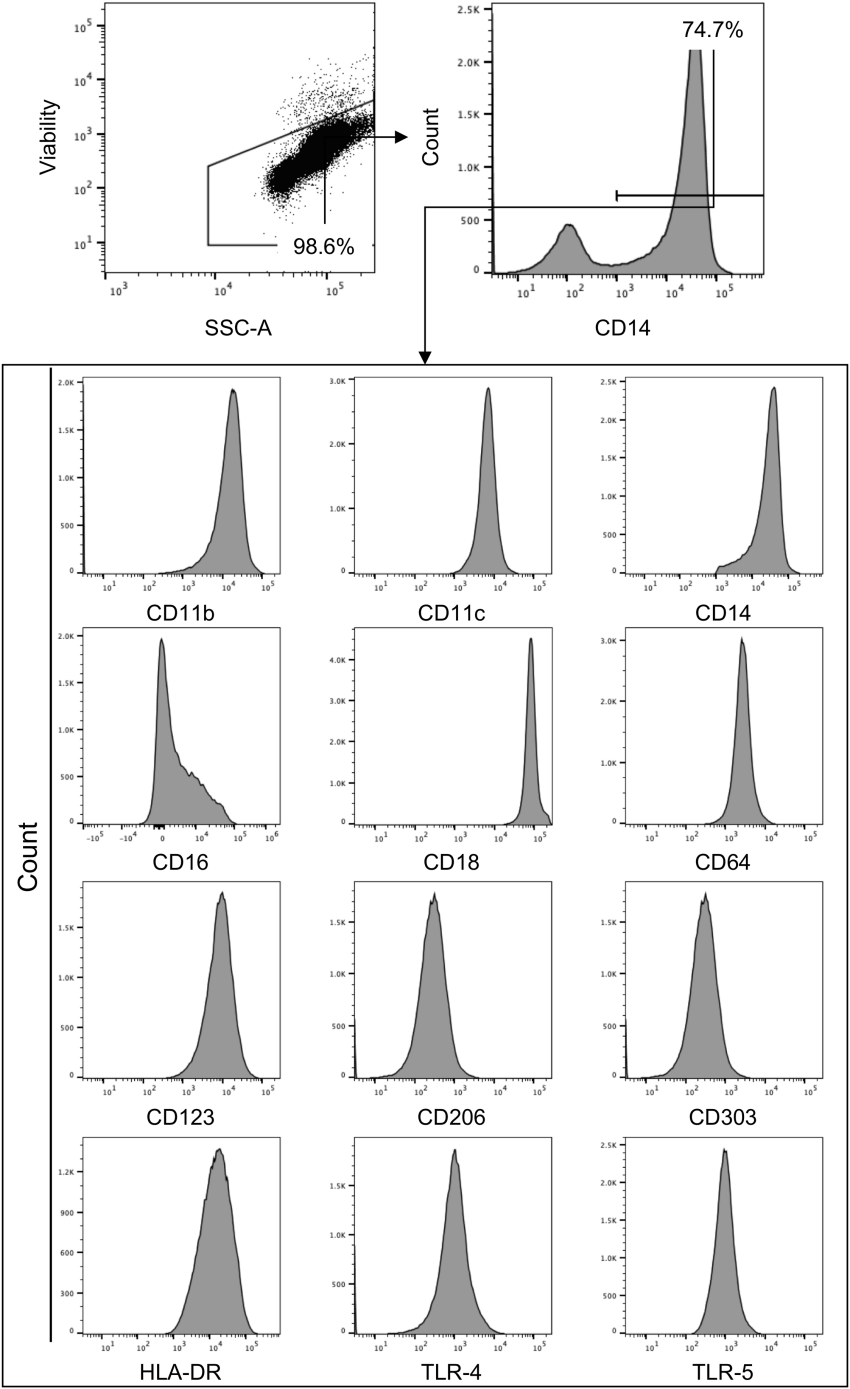
Representative flow cytometric gating strategy for phenotypic analysis of CD14^+^monocytes. Dot plots and histograms are shown for cells isolated from peripheral blood. Dead cells were removed by staining for viability (LIVE/DEAD) and gating on the negative population. Monocytes were identified according to the expression of CD14. The expression intensity of CD11b (integrin α_M_), CD11c (integrin α_X_), CD14, CD16 (FcRIII), CD18 (integrin β_2_), CD64 (FcγRI), CD123 (IL-3RA), CD206 (mannose receptor), CD303 (BDCA-2), HLA-DR, TLR-4, and TLR-5 was assessed.

We measured the relative expression intensity of CD11b (integrin α_M_), CD11c (integrin α_X_), CD14, CD16 (FcRIII), CD18 (integrin β_2_), CD64 (FcγRI), CD123 (IL-3RA), CD206 (mannose receptor), CD303 (BDCA-2), HLA-DR (human leukocyte antigen - DR isotype), TLR-4, and TLR-5 on the surface of nonstimulated CD14^+^ monocytes from vaccinated and unvaccinated volunteers by flow cytometry. A sequential gating strategy was used to identify populations of interest (Fig. 1). We compared the phenotypic properties of cells isolated from vaccinated and unvaccinated volunteers at day 0 with those isolated at 14 days, 3 months, and 6 months.

**Fig. 2:**
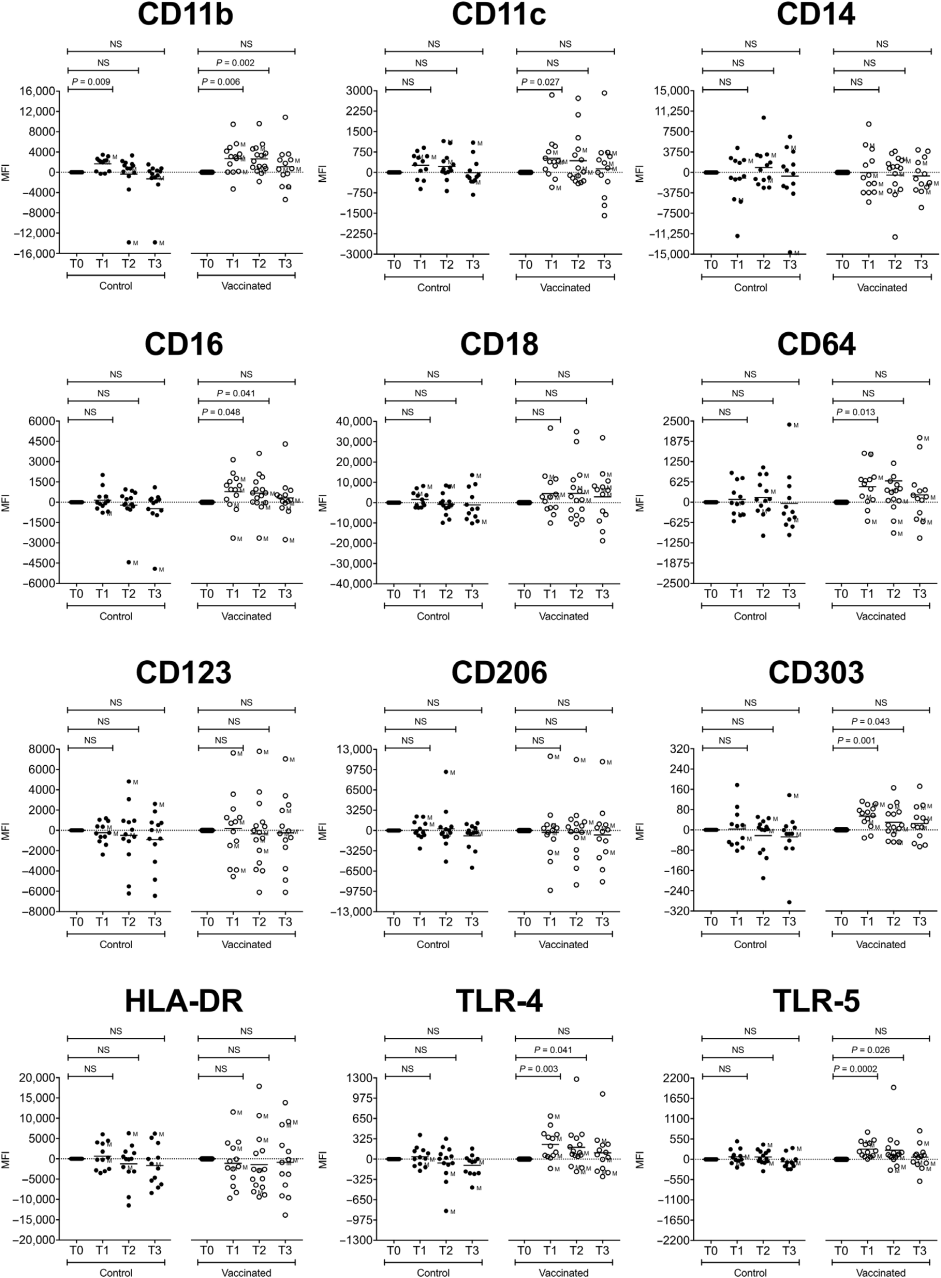
Change in surface receptor expression intensity among CD14^+^monocytes isolated from unvaccinated control group volunteers and volunteers vaccinated with *S.* Typhi strain Ty21a. The change in MFI of CD11b (integrin α_M_), CD11c (integrin α_X_), CD14, CD16 (FcRIII), CD18 (integrin β_2_), CD64 (FcγRI), CD123 (IL-3RA), CD206 (mannose receptor), CD303 (BDCA-2), HLA-DR, TLR-4, and TLR-5 among CD14^+^ monocytes isolated from control group volunteers (closed circles) and vaccinated volunteers (open circles). Samples were collected at day 0 (T0), 14 days (T1), 3 months (T2), and 6 months (T3). For each volunteer, the change in MFI at T1, T2, and T3 was calculated relative to T0. Statistical comparisons were performed on raw data. Horizontal bars represent mean values. NS, not significant; M, male volunteer.

We observed increased expression of CD11b, CD11c, CD16, CD64, CD303, TLR-4, and TLR-5 among vaccinated volunteers at 14 days (*P* = 0.006, *P* = 0.027, *P* = 0.048, *P* = 0.013, *P* = 0.001, *P* = 0.003, and *P* = 0.0002, respectively; Fig. 2). Increased expression of CD11b, CD16, CD303, TLR-4, and TLR-5 was observed at 3 months (*P* = 0.002, *P* = 0.041, *P* = 0.043, *P* = 0.041, and *P* = 0.026, respectively; Fig. 2). Expression of all receptors among vaccinated volunteers was comparable with baseline at 6 months. No change in the expression intensity of CD14, CD18, CD123, CD206, and HLA-DR was observed among the vaccinated group at any time point. Among control group volunteers, increased expression of CD11b was observed at 14 days (*P* = 0.009; Fig. 2). No change in the expression intensity of any other receptor at any time point was observed among the control group (Fig. 2).

Among vaccinated volunteers, where increased expression was observed at 14 days, men were fairly evenly distributed throughout the population; however, at 3 months, even where increased expression persisted, men tended to be clustered below the mean line (Fig. 2).

### Cytokine production

**Fig. 3:**
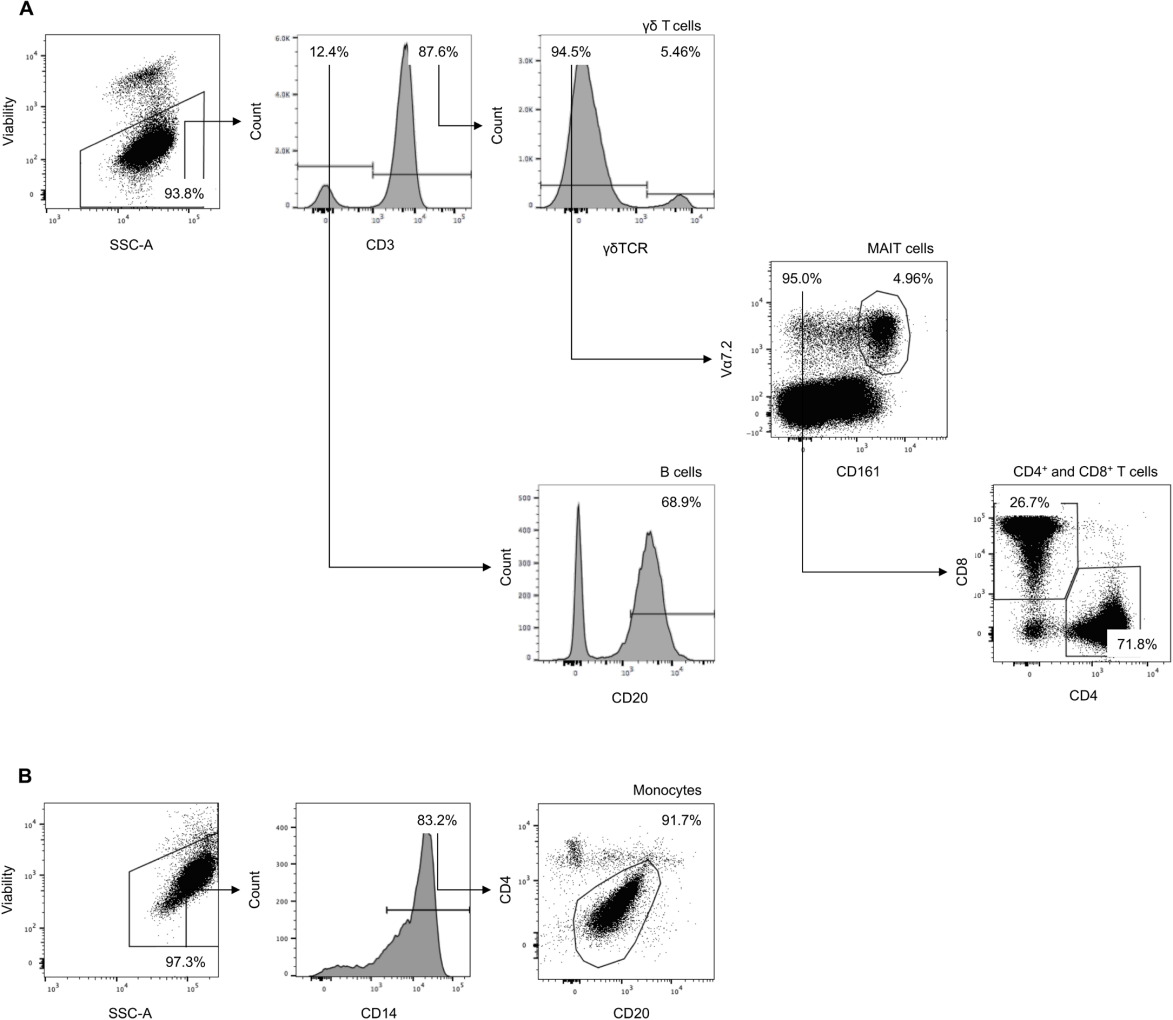
Representative flow cytometric gating strategy for intracellular cytokine analysis. Dot plots and histograms are shown for cells isolated from peripheral blood. The expression of IFN-γ, IL-4, IL-17A, TGF-β, and TNF-α was assessed in nonstimulated and in heat-killed live-attenuated *S.* Typhi strain Ty21a (Ty21a), heat-killed *C. albicans*, split-viron influenza (Influenza), PPD from *M. tuberculosis* (TB PPD), and tetanus toxoid–stimulated samples. (**A**) Dead cells were removed by staining for viability (LIVE/DEAD) and gating on the negative population. CD3^+^ subpopulations were defined as follows: γδ T cells were identified as cells that expressed the γδ T cell receptor, MAIT cells were identified as cells that expressed both CD161 and Vα7.2 that had not already been identified as γδ T cells, and CD4^+^ T cells and CD8^+^ T cells were identified as cells that expressed either CD4 or CD8 that had not already been identified as γδ T cells or MAIT cells. (**B**) Dead cells were removed by staining for viability (LIVE/DEAD) and gating on the negative population. Monocytes were identified according to the expression of CD14 and the intermediate expression of CD4 and CD20.

**Fig. 4:**
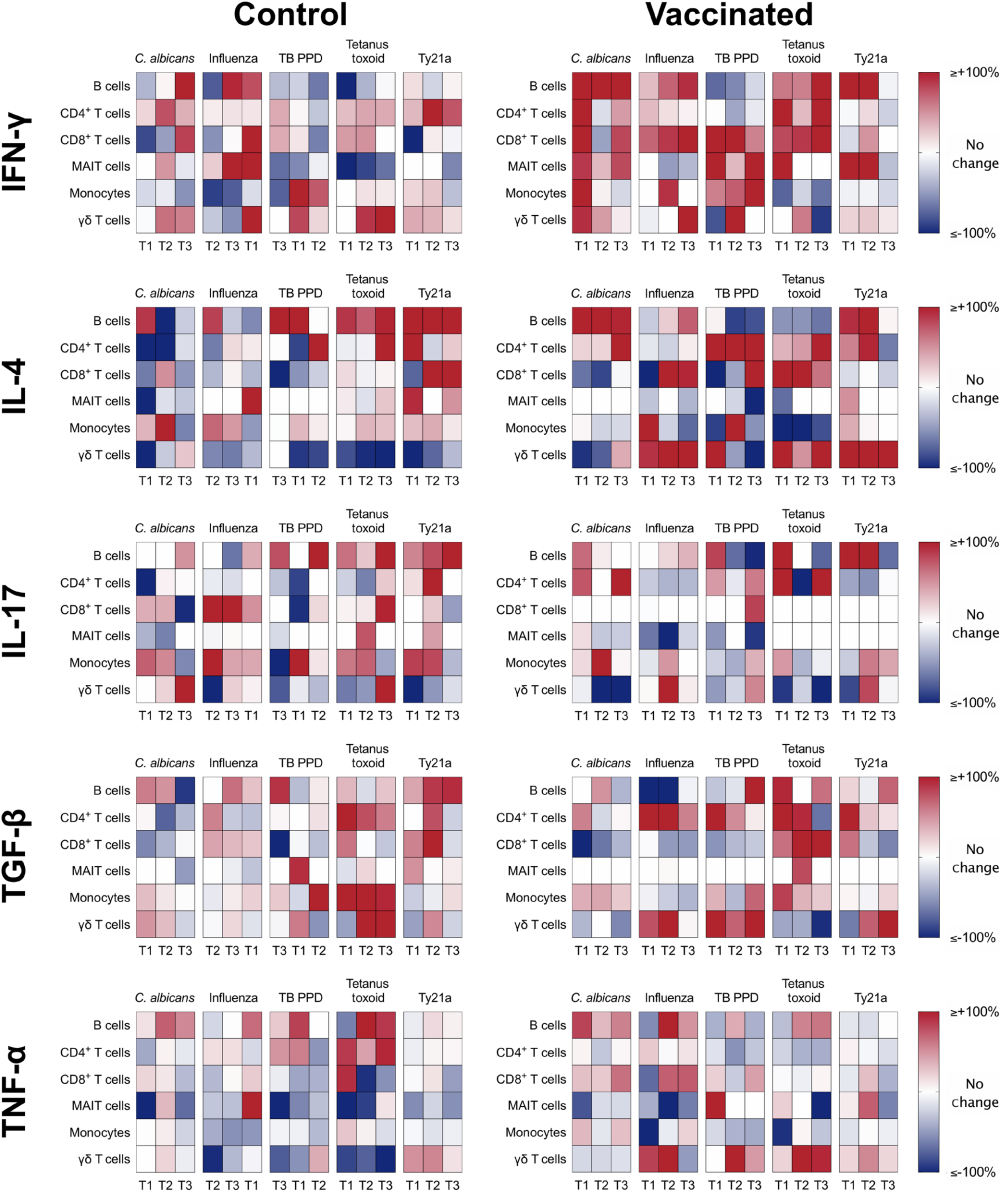
Heat map showing change in iMFI among unvaccinated control group volunteers and volunteers vaccinated with *S.* Typhi strain Ty21a. For each volunteer, change in iMFI (IFN-γ, IL-4, IL-17A, TGF-β, and TNF-α) was calculated at 14 days (T1), 3 months (T2), and 6 months (T3) relative to day 0 (T0). The median change in iMFI for each group was calculated and graphically represented using a double color gradient ranging from no change (white) to ≥+100% (red) and from no change (white) to ≤−100% (blue). Data were compiled for each of the following stimuli: heat-killed live-attenuated *S.* Typhi strain Ty21a (Ty21a), heat-killed *C. albicans*, split-viron influenza (Influenza), PPD from *M. tuberculosis* (TB PPD), and tetanus toxoid.

Cells were stimulated in vitro with a range of bacterial, fungal, or viral antigens: heat-killed live-attenuated *S*. Typhi strain Ty21a, heat-killed *Candida albicans*, split-viron influenza virus, purified protein derivative (PPD) from *Mycobacterium tuberculosis*, or tetanus toxoid. We then assessed the production of IFN-γ, IL-4, IL-17A, TGF-β, and TNF-α among B cells, CD4^+^ T cells, CD8^+^ T cells, monocytes, MAIT cells, and γδ T cells by flow cytometry. A sequential gating strategy was used to identify populations of interest (Fig. 3). The change in integrated mean fluorescence intensity (iMFI) at 14 days, 3 months, and 6 months was calculated relative to day 0. Heat maps showing change in iMFI were used to summarize data from vaccinated and unvaccinated volunteers (Fig. 4).

To compare the iMFI profiles of vaccinated and unvaccinated volunteers, we performed multivariate linear discriminant analysis of principal components (DAPC) to determine whether vaccination had influenced heterologous in vitro cytokine production. DAPC is particularly useful since it reduces the impact of variation within the groups.

**Fig. 5:**
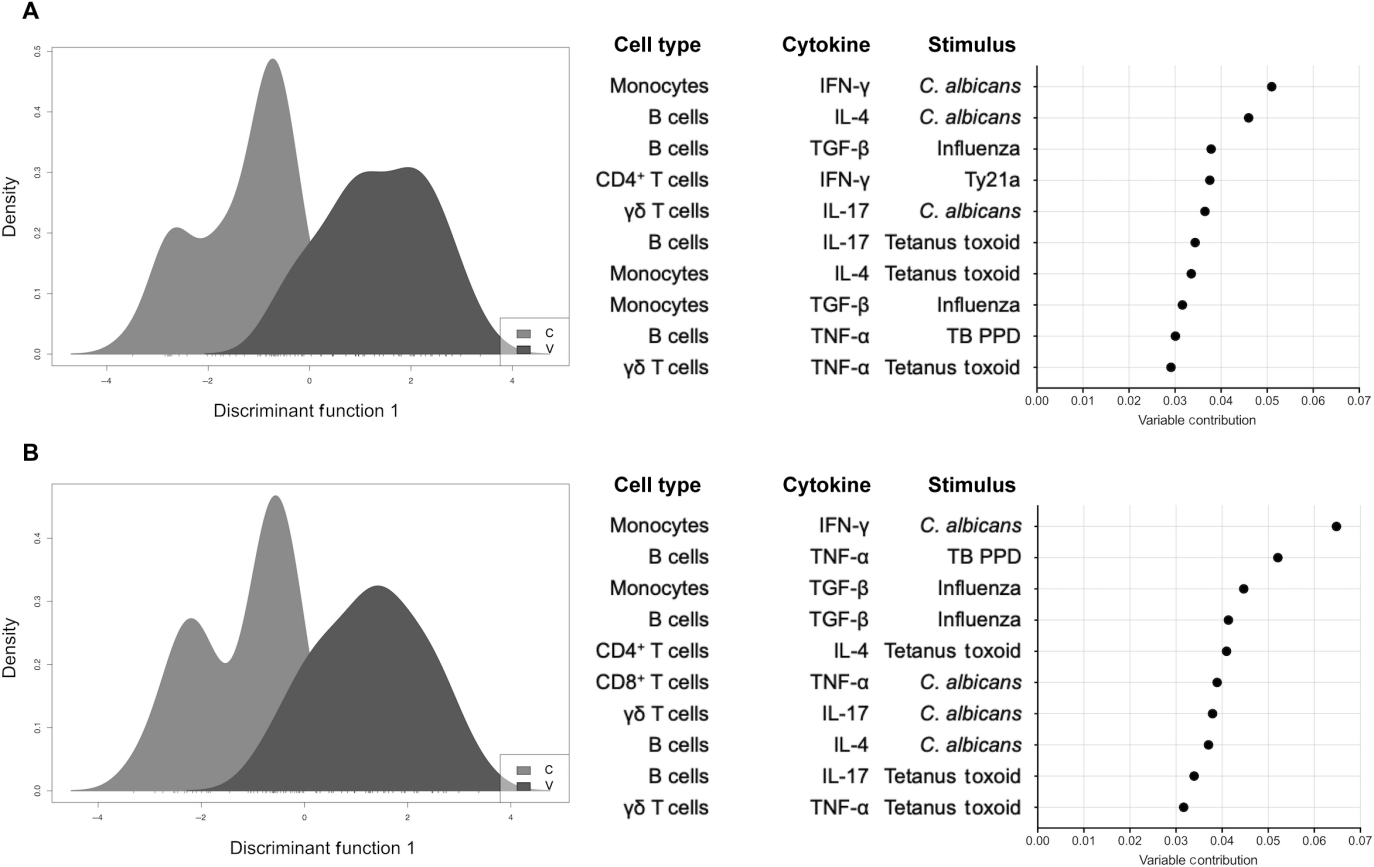
DAPC alongside the 10 largest contributing variables. For each volunteer, change in iMFI (IFN-γ, IL-4, IL-17A, TGF-β, and TNF-α) was calculated at 14 days (T1), 3 months (T2), and 6 months (T3) relative to day 0 (T0). Two models were built using data collected from control (C; light gray) and vaccinated (V; mid-gray) volunteers. (**A**) Data were compiled for the following stimuli: live-attenuated *S.* Typhi strain Ty21a, *C. albicans*, influenza virus, PPD from *M. tuberculosis*, and tetanus toxoid. (**B**) Data were compiled for the following stimuli: *C. albicans*, influenza virus, PPD from *M. tuberculosis*, and tetanus toxoid, with the notable exclusion of the live-attenuated *S.* Typhi strain Ty21a stimulus.

We first created a DAPC model that included all data collected for each stimulus, cell type, and cytokine and used it to predict volunteer vaccination status. This model, which comprised 24 principal components, was able to reclassify all volunteers with an accuracy of 92.4% (Fig. 5A). Loading plots (Fig. 5A), which detail the variables that contributed most toward the accuracy of DAPC, were combined with summary evidence from heat maps to identify changes in output that were most marked between vaccinated and unvaccinated volunteers (Fig. 4). These data revealed that the largest contributing variable was the monocyte IFN-γ responses to heat-killed *C. albicans* (increased output in the vaccinated group) and that the second largest contributing variable was the B cell IL-4 responses to heat-killed *C. albicans* (increased output in the vaccinated group).

When we created a second DAPC model, which excluded Ty21a stimulus data, the accuracy of the model, which also comprised 24 principal components, was unchanged and was able to reclassify all volunteers with an accuracy of 92.4% (Fig. 5B). Loading plots (Fig. 5B), combined with summary evidence from heat maps (Fig. 4), revealed that, once more, the largest contributing variable was the monocyte IFN-γ responses to heat-killed *C. albicans* (increased output in the vaccinated) and that the second largest contributing variable was the B cell TNF-α responses to PPD from *M. tuberculosis* (increased output in the control group and decreased output in the vaccinated group).

Visual inspection of heat maps also revealed a tendency toward increased IFN-γ among all cell types in response to heat-killed *C. albicans* in the vaccinated group. These data suggest that vaccination with Ty21a does influence responses to an array of unrelated pathogens and that vaccinated volunteers may be identified on the basis of these altered cytokine expression profiles.

## DISCUSSION

We have demonstrated that oral vaccination with *S*. Typhi strain Ty21a can induce up-regulation of CD11b, CD11c, CD16, CD64, CD303, TLR-4, and TLR-5 among CD14^+^ monocytes for as long as 3 months. We have further demonstrated that vaccination with Ty21a alters cytokine production among various cell populations in response to stimulation with nonrelated pathogens.

In studies assessing the nonspecific beneficial effects of vaccination, TLR-4, as well as CD14, CD11b, and CD206, has been shown to be up-regulated among monocytes for at least 1 year following vaccination with BCG (*13*). Challenge with wild-type *S*. Typhi has been shown to induce activation of circulating monocytes and dendritic cells (DCs) for up to 96 hours following onset of disease (*20*). Here, we have observed the increased expression of TLR-4 and TLR-5 on monocytes for at least 3 months following vaccination with Ty21a—this is likely to be a response to the engagement of these TLRs by lipopolysaccharide (LPS) and flagella, respectively. In addition to changes among TLRs, which are semipathogen specific, we also observed increased expression of CD11b, CD11c, CD16, CD64, and CD303; the up-regulation of these molecules is associated with enhanced phagocytic function and antigen presentation as well as enhanced chemotaxic potential (*21*–*23*). Consistent with published observations that demonstrate that nonspecific effects of vaccination are most marked in females (*9*, *24*–*26*), changes to monocyte phenotype among our study cohort appeared to be most long-lived among female volunteers. Unfortunately, since this was not an a priori hypothesis, and considerably more females were recruited to this study than males, we were unable to perform robust statistical analysis concerning the impact of gender, and further study would be required to confirm this important observation.

In murine models, live-attenuated influenza vaccine has been shown to confer immediate protection against respiratory syncytial virus (*27*). We have previously reported the early generation of cellular responses to influenza virus at the human duodenal mucosa 18 days following vaccination with Ty21a (*16*). These earlier nonspecific effects of vaccination, possibly a result of short-lived, localized, and self-limiting inflammation and/or up-regulation of cellular homing markers, may reduce susceptibility to infection in the days/weeks following vaccination, through heightened localized mucosal immune defense. It is significant, however, that here we have observed the increased expression of CD11b, CD16, CD303, TLR-4, and TLR-5 among monocytes for at least 3 months. Since monocytes typically only persist in circulation for a single day (*28*), we believe that the changes we have observed here are the result of epigenetic modifications occurring at the progenitor level. Consistent with this, treatment with LPS has been shown to phosphorylate the stress-response transcription factor ATF7, leading to reduced repressing histone methylation (H3K9me2) and increased activating histone methylation (H3K4me3), resulting in increased ATF7 target gene expression and enhanced pathogen resistance (*29*, *30*). Since no differences were observed here at 6 months, it is likely that monocytes do eventually return to a phenotype more in line with the individuals’ natural state. Nevertheless, in the 3 months following vaccination, these changes are likely to have a significant impact on the capture and presentation of antigen by differentiated monocytes—DCs and macrophages—and the adaptive immune responses that are subsequently generated.

Many factors influence human cytokine production; variation has been reported over a period as short as 24 hours (*31*). We observed temporal variation in cytokine production in the unvaccinated control group over a 6-month period. It is possible that changes in the control group may be the result of seasonal variation in the relative frequency with which different pathogens are encountered. With regard to our study cohort, the first sample from the first volunteer was acquired in May and the last sample from the last volunteer was acquired in December. To ensure that effects, particularly those surrounding heterologous responses, are not wrongly attributed to vaccination or clinical intervention, temporally matched controls such as those used here are essential. This is important since temporal variation may, at least in part, contribute toward altered cytokine production that has been observed elsewhere (*13*).

OPV, BCG, and measles containing vaccines appear to be associated with reductions in all-cause mortality (*3*–*7*). It has previously been demonstrated that cytokine production in response to in vitro stimulation with heterologous antigens is altered following vaccination with BCG (*13*). It is unclear whether changes in cytokine production are the result of altered antigen presentation occurring in vitro or whether these changes reflect modifications to immune cell population composition in vivo. To our knowledge, this is the first instance where the simultaneous assessment of cytokine production by multiple cell types has been attempted in the context of trained immunity, with previous studies having relied on the assessment of supernatants collected following in vitro stimulation (*13*).

Our use of DAPC has allowed us to make the general conclusion that vaccination with Ty21a does alter cytokine production to nonrelated pathogens. Since DAPC modeling revealed a similar capacity to reassign volunteers, even when Ty21a data were excluded, we can assume that the specific responses generated to the vaccine were not the only responses that enabled discrimination of the two groups. This suggests that vaccination with Ty21a did alter cytokine responses to heterologous antigens. Variation within the human population may mean that we are underpowered to observe some other important biological effects.

It is notable that responses to heat-killed Ty21a were not the largest contributors to DAPC. Rather, we observed that monocyte IFN-γ responses to heat-killed *C. albicans* and B cell IL-4 responses to heat-killed *C. albicans* contributed most significantly to DAPC. These responses were increased in the vaccinated group, and since IL-4 is essential for the development of protective T_H_1 (IFN-γ) responses to *C. albicans* (*32*), the data we have presented would suggest that volunteers vaccinated with Ty21a may be less susceptible to infection by *C. albicans*. Monocyte and B cell responses to a range of other antigens, including those derived from *M. tuberculosis* and influenza, consistently featured among the largest contributors to DAPC. Differentiated monocytes—DCs and macrophages—play a prominent role in innate immune defense and in antigen presentation. B cells, in addition to their role in immunoglobulin production, also play a role in antigen presentation and in the generation of T cell responses (*33*, *34*). Data presented here would suggest that modified cytokine output by monocytes and B cells, cells that also play a role in antigen presentation, may be particularly important with regard to the generation of the nonspecific beneficial effects that have been observed elsewhere (*3*–*7*). It should be noted that, to date, no data have been presented that demonstrate that these kinds of immunological observations are responsible for observed reductions in all-cause mortality in humans (*35*). Further study is warranted to more fully characterize the contribution of innate, proinflammatory, and regulatory cell types and effector molecules in conferring these nonspecific beneficial effects.

The burden of typhoidal disease is highest in children under 5 years (*36*). Although Ty21a, in its currently licensed capsule formulation, is not approved for use in children, it has previously been demonstrated that, when administered in liquid suspension, Ty21a is fully immunogenic in children aged between 2 and 6 years (*37*, *38*). The emergence and spread of antimicrobial-resistant *S.* Typhi (*39*) and the more recent emergence of extensively drug-resistant *S.* Typhi (*40*) have placed increased importance on the development of more effective vaccines targeting *S.* Typhi, which may be easily administered in regions of endemic disease. In addition to their direct impact on typhoid, it is possible that next-generation orally administered live-attenuated *Salmonella* vaccines, which are at various stages of development (*41*), may also have indirect beneficial effects.

Data presented here support the development of orally administered live-attenuated *Salmonella*-based adjuvant/vector platforms that may confer nonspecific beneficial effects. Ty21a, or other live-attenuated *Salmonella* vaccines, may also have direct applications in their own right as interventions capable of reducing all-cause mortality. OPV—a vaccine that has been shown to reduce all-cause mortality by as much as 17% (*7*)—will soon be replaced by inactivated polio vaccine (IPV). While IPV does not carry the same risk of vaccine-associated paralytic poliomyelitis, the replacement of OPV with IPV may actually have a detrimental impact on all-cause mortality owing to the inability of IPV to confer the nonspecific beneficial effects that have previously been associated with OPV (*42*, *43*). It is possible that vaccines such as Ty21a, which are low cost, extremely well tolerated, and easily administered, could be included in wider vaccination programs, even in non–typhoid-endemic regions, solely for their off-target, nonspecific beneficial effects. Since it has been demonstrated that a liquid formulation of Ty21a is immunogenic in children (*37*, *38*), this strategy could potentially be tested for relevance in the field in young age groups, where its impact would likely be greatest. If this strategy were to be pursued, it would represent a step change in global vaccination policy and could have a profoundly positive impact on populations in low-income settings.

## MATERIALS AND METHODS

### Ethical approval, recruitment, and study protocol

All volunteers provided written informed consent. This study was approved by the United Kingdom National Research Ethics Service (14/NW/1455). Thirty healthy adult volunteers were enrolled into the study. Sixteen volunteers (4 males and 12 females) were randomly selected for oral vaccination with live-attenuated *S*. Typhi (Ty21a; Vivotif). Three doses of a single oral capsule were taken on days 0, 2, and 4, approximately 1 hour before a meal, with a cold or lukewarm drink. Fourteen volunteers (2 males and 12 females) were randomly assigned to an unvaccinated control group.

### Peripheral blood mononuclear cell isolation

Peripheral blood samples were collected in lithium heparin Vacutainers at day 0, 14 days, 3 months, and 6 months. Peripheral blood mononuclear cells (PBMCs) were isolated via Ficoll-Paque PLUS (GE Healthcare) using LeucoSep Centrifuge Tubes (Greiner), according to the manufacturer’s instructions. Full details are presented in Supplementary Materials and Methods.

### Antigenic stimulation and incubation

PBMCs (1 × 10^6^ cells per well) were seeded in complete medium in 96-well V-bottom plates. Cells in each well were stimulated with either 2 × 10^6^ colony-forming units (CFU) of heat-killed *S.* Typhi Ty21a (Vivotif; suspended in Dulbecco’s PBS, quantified, and then killed by incubation at 95°C for 30 min), 2 × 10^6^ CFU of heat-killed *C. albicans* (quantified and then killed by incubation at 56°C for 30 min), 5 μg of PPD from *M. tuberculosis* (TB PPD), 20 μg of tetanus toxoid, or 180 ng of hemagglutinin from, in equal quantities, an A/California/7/2009 H1N1-like strain, an A/Texas/50/20122 H3N2-like strain, and a B/Massachusetts/2/2012-like strain. One negative control well was treated with complete medium to adjust for non–antigen-specific background cytokine production. Cells were then incubated at 37°C in 5% CO_2_. After 2 hours, 1 μl of brefeldin A (BD GolgiPlug; BD Biosciences) and 1 μl of monensin (BD GolgiStop; BD Biosciences) were added to each well, and the plate was incubated for a further 16 hours at 37°C in 5% CO_2_.

### Flow cytometric analyses

For phenotypic analysis, PBMCs were washed before being stained for viability and surface phenotype. For intracellular cytokine analysis, PBMCs were washed, stained for viability and surface phenotype, and, following fixation and permeabilization, stained for intracellular cytokine production.

Details of the antibodies that were used are presented in Supplementary Materials and Methods. Cells were washed, resuspended, and stored in the absence of light at 4°C until data were acquired using a FACSAria III flow cytometer (BD Biosciences). Compensation beads (BD Biosciences) were used to create compensation matrices, and sequential cell isolation was used to identify populations of interest (Figs. 1 and 3). For phenotypic analysis, values were expressed as the median fluorescence intensity. For intracellular cytokine analysis, the frequency of each population positive for each cytokine above background was multiplied by the geometric MFI for each cytokine-positive population—this well-established metric is known as the iMFI (*44*). Full details are presented in Supplementary Materials and Methods.

### Statistical analyses

Paired comparisons were made using Wilcoxon matched-pairs signed-rank test using Prism v6 (GraphPad). *P* values are two-tailed and considered significant at *P* < 0.05.

Linear DAPC models were used to predict volunteer vaccination status. DAPC models were created using the package adegenet (*45*) within R (*46*). The optimal number of principal components for each model was calculated via cross-validation. Cross-validation split the data into a training set (comprising 90% of the data) and a validation set (comprising 10% of the data). The validation set was changed through random sampling and was classified by the model produced from the training set. This procedure was repeated 1000 times for every number of retained principal components, and the lowest number of principal components with the highest proportion of correct predictions was selected. This resulted in 24 principal components being retained for each model.

## Supplementary Material

http://advances.sciencemag.org/cgi/content/full/5/2/eaau6849/DC1

## SUPPLEMENTARY MATERIALS

Supplementary material for this article is available at http://advances.sciencemag.org/cgi/content/full/5/2/eaau6849/DC1 Supplementary materials and methods
