# Supplementary material for: Nonspecific effects of oral vaccination with live-attenuated Salmonella Typhi strain Ty21a
Source: Sci Adv. 2019 Feb 27;5(2):eaau6849. doi: 10.1126/sciadv.aau6849 (PMC6392763; doi:10.1126/sciadv.aau6849)
Supplement: http://advances.sciencemag.org/cgi/content/full/5/2/eaau6849/DC1 [file supp_5_2_eaau6849__index.html]

Science Advances | Science Advances

## Supplementary Materials

**This PDF file includes:**

- Supplementary materials and methods

Download PDF

**Files in this Data Supplement:**

- Adobe PDF - aau6849\_SM.pdf
