# Supplementary material for: Nonspecific effects of oral vaccination with live-attenuated Salmonella Typhi strain Ty21a
Source: Sci Adv. 2019 Feb 27;5(2):eaau6849. doi: 10.1126/sciadv.aau6849 (PMC6392763; doi:10.1126/sciadv.aau6849)
Supplement: http://advances.sciencemag.org/cgi/content/full/5/2/eaau6849/DC1 [file aau6849_SM.pdf]

Science Advances
AAAS

advances.sciencemag.org/cgi/content/full/5/2/eaau6849/DC1

Supplementary Materials for

Nonspecific effects of oral vaccination with live-attenuated *Salmonella* Typhi strain Ty21a

S. H. Pennington*, D. M. Ferreira, E. Caamaño-Gutiérrez, J. Reiné, C. Hewitt, A. D. Hyder-Wright, S. B. Gordon, M. A. Gordon*

*Corresponding author. Email: shaun.pennington@lstmed.ac.uk (S.H.P.); magordon@liverpool.ac.uk (M.A.G.)

Published 27 February 2019, *Sci. Adv.* **5**, eaau6849 (2019)
DOI: 10.1126/sciadv.aau6849

This PDF file includes:

Supplementary Materials and Methods

Supplementary Materials and Methods

Peripheral blood mononuclear cell (PBMC) isolation

Peripheral blood samples were collected in lithium heparin Vacutainers (BD Biosciences). Samples were layered on Ficoll-Paque PLUS using LeucoSep centrifuge tubes and centrifuged at 800 g for 18 minutes at room temperature without brake. Cells were isolated using a sterile Pasteur pipette, washed twice (centrifuged at 250 g for 10 minutes) with $HBSS^{-/-}$ and resuspended in 10mL $HBSS^{-/-}$. A sample of the cell suspension was diluted 1:1 by adding 10 µL of the cell suspension to 10 µL 0.4% Trypan Blue. The number of viable cells was then enumerated.

Cells were stored using CTL-Cryo ABC media. Cells were washed once (centrifuged at 250 g for 10 minutes) with $HBSS^{-/-}$ and the cell pellet resuspended in CryoC at a concentration of 2×10^7 cells/mL. Samples were then placed on ice and an equal volume of CryoA:CryoB (4:1) was slowly added to achieve a final concentration of 1×10^7 cells/mL. The cell suspension was aliquoted into cryovials in 1 mL volumes. Cryovials were then placed in a cooled Mr. Frosty freezing container and transferred to -80˚C. After 24 hours cryovials were transferred on dry ice to -200˚C storage.

Cells were removed from -200˚C storage and incubated at 37°C for 10 minutes. Cryovials were inverted twice and the contents of the cryovial transferred into 15 mL Falcon tubes. Cryovials were washed once with 1 mL prewarmed DNase I medium in order to collect any remaining cells. An additional 8 mL prewarmed DNase I medium was added to the 15 mL Falcon tubes at a rate of 1 mL every five seconds. Samples were washed twice (centrifuged at 330 g for 10 minutes) with prewarmed DNase I medium and resuspended in 600 µL prewarmed complete medium. A sample of the cell suspension was diluted 1:1 by adding 10 µL of the cell suspension to 10 µL 0.4% Trypan Blue. The number of viable cells was then enumerated.

The concentration of the cell suspension was adjusted with complete medium to 1×10^7 cells/mL. 100 µL of the cell suspension was then added to each well of a 96-well plate, as required. Cells were then 'rested' overnight at 37°C in 5% CO_2. The following day, plates were centrifuged at 330 g for 10 minutes, the supernatant removed and the cells in each well resuspended in 100 µL prewarmed complete medium.

Flow cytometric analyses

For phenotypic analysis, PBMCs were washed and stained for viability (Vivid®; Invitrogen) and surface phenotype using antibodies specific to CD14-PerCP/Cy5.5 (MφP9; BD Biosciences), CD16-APC/Cy7 (FcγRIII; BioLegend), CD64-BV605 (HI10a; BioLegend), CD18-PE/Cy7 (TS1/18; BioLegend), CD11b-AF700 (ICRF44; BD Biosciences), CD11c-Pacific Blue (Bu15; BioLegend), CD123-BV711 (6H6; BioLegend), CD206-PE/CF594 (19.2; BD Biosciences), CD284-APC (HTA125; BioLegend), CD285-FITC (624915; R&D Systems), CD303-PE (201A; BioLegend) and CD161-BV785 (L243; BioLegend). Cells were washed and stored in the absence of light at 4°C until data were acquired using a FACSAria III flow cytometer (BD Biosciences).

For intracellular cytokine analysis, following stimulation and incubation, PBMCs were washed and stained for viability (Vivid®; Invitrogen) and surface phenotype using antibodies specific to CD3-APC/H7 (SK7; BD Biosciences), CD4-PerCP/Cy5.5 (SK3; BioLegend), CD8-BV650 (SK1; BioLegend), CD14-APC/H7 (MφP9; BD Biosciences), CD20-Pacific Blue (2H7;

BioLegend), CD161-BV785 (HP-3G10; BioLegend), TCRγδ-PE/Cy7 (11F2; BD Biosciences) and Vα7.2-PE/Dazzle594 (3C10; BioLegend). Following fixation and permeabilisation (Cytofix/Cytoperm™; BD Biosciences), cells were stained for intracellular interferon (IFN)-γ-PE (4S.B3; BioLegend), tumour necrosis factor (TNF)-α-FITC (MAb11; BioLegend), interleukin (IL)-17A-AF700 (N49-653; BD Biosciences) and IL-4-BV711 (MP4-25D2; BD Biosciences) and transforming growth factor (TGF)-β-APC (TW4-6h10; BioLegend). Cells were washed in Perm/Wash™ (BD Biosciences), resuspended in CellFIX™ (BD Biosciences), and stored in the absence of light at 4°C until data were acquired using a FACSAria III flow cytometer (BD Biosciences).

Compensation beads (BD Biosciences) were used to create compensation matrices and sequential cell isolation used to identify populations of interest (Fig. 1 and Fig. 3). IFN-γ^{+}, TNF-α^{+}, IL-17A^{+}, IL-4^{+} and TGF-β^{+} populations were positively identified using FlowJo version 10 (Treestar Inc.).
